# Determining the Efficiency of the Sponge City Construction Pilots in China Based on the DEA-Malmquist Model

**DOI:** 10.3390/ijerph191811195

**Published:** 2022-09-06

**Authors:** Heng Zhang, Qian Chang, Sui Li, Jiandong Huang

**Affiliations:** 1 School of Management Science and Engineering, Anhui University of Finance and Economics, Bengbu 233030, China; 2 School of Information Management, Central China Normal University, Wuhan 430079, China; 3 School of Statistics and Applied Mathematics, Anhui University of Finance and Economics, Bengbu 233030, China; 4 School of Civil Engineering, Guangzhou University, Guangzhou 510006, China; 5 School of Mines, China University of Mining and Technology, Xuzhou 221116, China

**Keywords:** sponge city construction, data envelopment analysis, efficiency evaluation, water ecological environment

## Abstract

Sponge city construction (SCC) has improved the quality of the urban water ecological environment, and the policy implementation effect of SCC pilots is particularly remarkable. Based on the data envelopment analysis (DEA) model, this study employed the related index factors such as economy, ecology, infrastructure, and the population of the pilot city as the input, and the macro factors of SCC as the output, to scientifically evaluate the relative efficiency between the SCC pilots in China. Eleven representative SCC pilots were selected for analysis from the perspectives of static and dynamic approaches, and comparisons based on the horizontal analysis of the efficiency of SCC pilots were conducted and some targeted policy suggestions are put forward, which provide a reliable theoretical model and data support for the efficiency evaluation of SCC. This paper can be used as a reference for construction by providing a DEA model for efficiency evaluation methods and thus helps public sector decision makers choose the appropriate construction scale for SCC pilots.

## 1. Introduction

With the advancement of China’s urbanization [1,2,3], water-related problems are worsening. In such circumstances, sponge city construction (SCC) is regarded as an innovative step in terms of green transformation, resilient construction, and low-carbon development of urban drainage management, considering due responsibility and aiming for flexibility in dealing with the natural disasters caused by urban environmental changes and water imbalances [4,5,6,7]. In 2015 and 2016, the Ministry of Finance, the Ministry of Housing and Urban–Rural Development, and the Ministry of Water Resources of China set up two batches of 30 typical national SCC pilots [8]. With the construction of these SCC pilots, the ability to resist rain and flood disasters, to control water pollution, and to improve the water environment has been strengthened. The pilots have been implemented with remarkable results. During the 14th Five-Year Plan Period (2021–2025), these three ministries and commissions will undertake the job of promoting SCC systematically and comprehensively [9,10]. Therefore, the construction of a scientific and comprehensive evaluation system is an important guarantee of the effectiveness of SCC pilots and the systematic promotion of SCC in the whole region [11,12,13]. The “Sponge City Construction Evaluation Standards” can ensure the construction quality, the performance level of SCC, and the overall direction of construction projects, but fail to reflect the internal relationship between the background and the construction level of sponge cities, as well as the efficiency of the SCC pilots.

Several scholars have conducted studies from different perspectives on the performance and efficiency of SCC evaluation. At the macro level, most scholars took the influencing factors involved in SCC as the evaluation index and constructed evaluation systems, considering the aspects of water environment, security, ecology, and resources by establishing various evaluation models [14,15,16]. Other researchers evaluated SCC via actual case data from pilots [17,18,19]. At the micro level, SCC evaluation is mainly based on sponge-body technology models such as the storm water management model (SWMM) [14,20]. Through model parameter calibration and scenario simulation, the effectiveness of the low-impact development (LID) construction of the sponge body is evaluated [21,22]. Compared with a macro evaluation, the index data of micro evaluation are difficult to collect, and massive monitoring facilities are needed to build the evaluation system for the whole city. Although the system for micro evaluation is more detailed and accurate, time and experience of data acquisition and technical means are still required. The macro evaluation of SCC is relatively simple and effective, directly reflecting the construction level of the sponge city through macro data. Additionally, from both macro and micro perspectives, the evaluation of SCC lacks horizontal comparison and efficiency evaluation between pilot cities. The efficiency evaluation of SCC incorporates different input factors such as urbanization level, urban population, and urban economic level, which may determine the production scale of SCC (urban water environment status). By comparing the same input of pilot cities, the output level can be obtained. When the output is the same, the lower the input, the higher the efficiency. For now, quantitative research is limited to a single-dimensional performance index, with little consideration of basic urban data on the economy, ecology, population, and other factors involved in SCC pilots [23]. It should be noted that these factors have a great impact on the construction and effectiveness of sponge cities. The geographical background and socioeconomic development of Chinese SCC pilots are complex and diverse. Though adopting the same capital and technical investment, different pilots may have different efficiency in SCC. Therefore, the quantitative results of basic indicators can provide a necessary basis for horizontal and relative comparisons between cities.

In summary, existing studies have mostly focused on a certain region or pilot city to evaluate the effectiveness of SCC, while few scholars have paid attention to efficiency evaluation. In addition, few studies have conducted horizontal efficiency evaluation among SCC pilots. It is necessary to study the differences in SCC efficiency between different pilots [24]. To evaluate the efficiency of SCC scientifically and objectively, this study constructed an SCC efficiency evaluation index model, based on data envelopment analysis (DEA), considering the related indicators of the city’s economy, ecology, infrastructure, population, and other factors as the input, and the macro indicators of the efficiency of SCC as the output [25]. As far as current research on the efficiency of SCC is concerned, most studies take capital investment in SCC projects as the basis, rarely considering factors such as the ecological foundation and population of the city, and the input factors are relatively simple. In addition, it is worth noting that research on SCC in China is mainly related to the dimensions of water resources, water ecology, water environment, and water safety (rain and flood management). However, it is difficult to obtain index data for the stormwater management dimension from the perspective of whole pilot cities; therefore, this paper mainly examines the efficiency of the SCC pilots from the perspective of the water environment. It provides new research ideas and theoretical models for quantitative evaluation and adaptive construction in the future.

**Figure 1:**
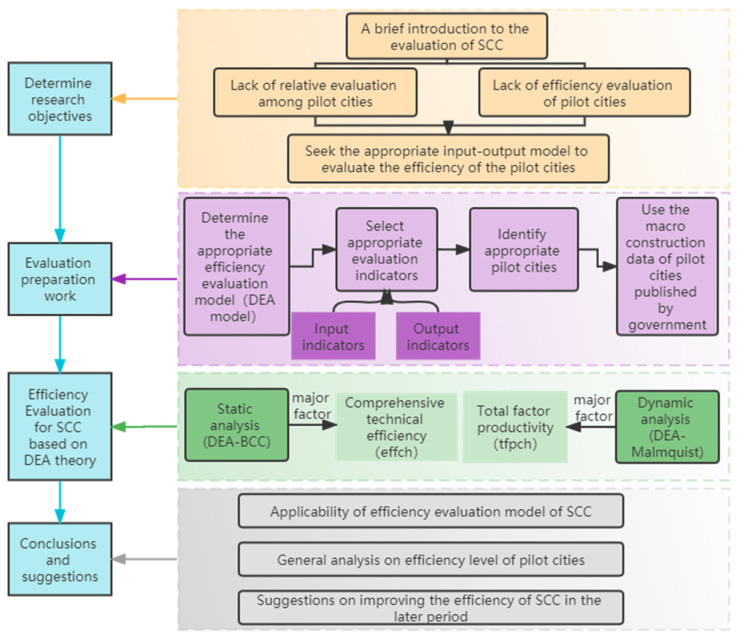
The efficiency evaluation framework of SCC pilots.

To sum up, a DEA theory-based efficiency measurement model of China’s SCC was constructed. The subsequent sections of this study are as follows. The model and data are presented in Section 2. The DEA static and dynamic models are discussed in Section 2.1, which introduces basic DEA theory. The efficiency evaluation indicators’ system of SCC is established in Section 2.2, which elaborates the input and output indicators. Suitable pilot cities are selected in Section 2.3, which describes the current situation and data sources. The efficiency evaluation of China’s SCC pilots based on the DEA-BCC-Malmquist model is presented in Section 3, which analyzes the results from static and dynamic perspectives. Section 4 summarizes the analysis and puts forward relevant suggestions. The efficiency evaluation framework of this study is given in Figure 1.

## 2. Model and Data

### 2.1. Basic DEA Model

A systematic study of efficiency theory was conducted by Farrell in 1957 [26]. Efficiency evaluation is always expressed as the ratio of an objective’s outputs to inputs at a specific time, from the perspective of management and economics, and has been studied and applied in different fields [27,28]. A DEA model is a specific model of data envelopment analysis, which analyzes the technical efficiency and scale efficiency of decision-making units (DMUs). It is based on objective data, not affected by the data dimension or other subjective factors. The DEA model possesses strong reliability and a scientific nature [29]. DEA is widely used in the economic management field, mainly for environmental efficiency analysis [30,31], green development efficiency assessment [32], eco-efficiency evaluation [33,34,35], energy efficiency [36], financial industry and innovation efficiency performance assessment [37,38], water use efficiency and flood vulnerability assessment [25,39,40], urban efficiency evaluation, etc. It has significant advantages in terms of simplifying calculations and processing multiple input–output indicators. The multiobjective evaluation of the DEA model could be used in the efficiency evaluation of SCC. Due to the multiple inputs and outputs of sponge city indicators, the model can make greater use of index data to analyze the efficiency and make horizontal comparisons between pilots.

#### 2.1.1. DEA Static Model

The traditional DEA model measures the relative efficiency and obtains the corresponding frontiers function, based on known data, to evaluate the efficiency of DMUs with multiple inputs and outputs. The efficiency measurement of Constant Returns to Scale (CRS) and Variable Returns to Scale (VRS) in DEA would be used to evaluate SCC input–output efficiency in China. To measure the construction efficiency of pilots and its influencing factors, the Banker, Charnes, and Cooper (BCC) model, with the assumption of variable returns to scale, was selected to analyze the technical and scale efficiency for the efficiency evaluation system of SCC pilots, as shown in the following equation [28]:

(1) $$\begin{array}{c} \mathrm{Min}\gamma \\ s.t.\left\{\begin{array}{l} \sum_{j=1}^{n}x_{j}\lambda_{j}+s^{-}=\gamma x_{0}\\ \sum_{j=1}^{n}y_{j}\lambda_{j}-s^{+}=y_{0}\\ \sum_{j=1}^{n}\lambda_{j}=1\\ \lambda_{j}\geq 0,s^{+}\geq 0,s^{-}\geq 0 \end{array}\right. \end{array}$$

The meanings of the symbols in Equation (1) are as follows. ‘s.t.’ is a fixed term of linear programming method, which means ‘subject to’. The present research selects *n* (*n* = 11) regions as DMUs, assuming the input *x*_0_ and output *y*_0_ are constant; *n* is the number of DMUs; *j* is the indexes for DMUs; *x_j_* is the input of the jth DMU, and *y_j_* is the output of the *j*th DMU (see Table 1 for specific input and output indicators); λ*_j_* is the corresponding weight coefficient; *γ* is the efficiency for the DMUs and the value range is [0,1]; *s^−^* is a relaxation variable representing the redundancy of input; and *s^+^* is a residual variable, while representing the insufficiency of output [30,41].

The results of the BCC model include comprehensive technical efficiency (Effch), pure technical efficiency (Pech), scale efficiency (Sech), and Effch = Pech × Sech. Comprehensive technical efficiency is an evaluation of DMU’s resource allocation ability, resource utilization efficiency, and other capabilities; pure technical efficiency refers to the production efficiency of DMUs affected by management and technology, and scale efficiency reflects the matching degree between the inputs and outputs of SCC [41,42]. When *γ* = 1, *s^−^* = 0, and *s^+^* = 0, the technical efficiency and scale efficiency of the DMUs are effective, the input and output of DMUs are not redundant or insufficient, and the overall DEA is effective.

When *γ* = 1 and *s^−^* ≠ 0 or *s^+^* ≠ 0, the technical efficiency or scale efficiency of the DMUs is invalid, and the DEA of the DMUs is weakly efficient. When *s^−^* ≠ 0, DMUs have input redundancy, and the scale efficiency of DMUs is invalid; the original output can be kept unchanged by reducing the input.

When *γ* = 1, the technical efficiency and scale efficiency of the DMUs are invalid. In that case, the DEA of the DMUs is invalid.

#### 2.1.2. DEA Dynamic Model

The BCC model is a traditional DEA model, which performs a static analysis of DMUs’ efficiency. In terms of dynamic analyses, the Malmquist index model can measure the dynamic efficiency of time series data and is now widely used in major research fields. The Malmquist model is based on DEA and calculates the input–output efficiency by the ratio of distance function [42,43]. The Malmquist index method calculates the Malmquist total factor productivity index (Tfpch) via the change in productivity from this period (*t*) to the next (*t* + 1), and makes a dynamic analysis of the DMUs’ efficiency. The calculation formula of the total factor productivity index is as follows [32]:

(2) $$\begin{array}{l} \mathrm{Effch}=\mathrm{Pech}\;\times \;\mathrm{Sech}\\ \mathrm{Tfpch}=\mathrm{Effch}\;\times \;\mathrm{Techch} \end{array},$$

where Pech represents the pure technical efficiency index of the pilot, which reflects the production efficiency of the input factors of the DMU at a certain scale (optimal scale); Sech represents the scale development efficiency index of the pilot, which reflects the difference between the actual scale and the optimal production scale; and Effch represents the comprehensive technical efficiency change index of the pilot, which is composed of two parts: Pech and Sech. Pech measures the gap (the management level of DMUs) between the actual technical efficiency and the benchmark technical efficiency, in the implementation of SCC pilots, while Sech measures the gap (the investment scale) between the actual technical scale and the optimal technical scale [41]. Techch represents the technological progress efficiency index of a pilot, while Tfpch represents the total factor productivity index of a pilot.

All the figures are taken to have a reference value of 1, representing an increase of more than 1, and a decrease of less than 1. The exponential method achieves effective decomposition of Tfpch by constructing a distance function. The distance function of Malmquist index *D^t^* and the Malmquist index from *t* to *t* + 1 are as follows. *D^t^*(*x^t^*, *y^t^*) and *D^t^*^+1^(*x^t^*, *y^t^*) are the distances between the DMU(*x^t^*, *y^t^*) in period *t* and the frontiers of period *t* and period *t* + 1, respectively. *D^t^*^+1^(*x^t^*^+1^, *y^t^*^+1^) and *D^t^*(*x^t^*^+1^, *y^t^*^+1^) are the distances between the DMU(*x^t^*^+1^, *y^t^*^+1^) in period *t* + 1 and the frontiers of period *t* + 1 and period *t*, respectively [27].

According to Equation (2) of the Tfpch, the Malmquist index takes the geometric average from Tfpch of the subsequent period. We can see the Malmquist index from year *t* to year *t* + 1 in Equation (3) [32]:

(3) $$M_{i}(x^{t+1},y^{t+1};x^{t},y^{t})=\mathrm{Tfpch}=\mathrm{Effch}\;\times \;\mathrm{Techch}=\frac{D_{i}^{t+1}(x^{t+1},y^{t+1})}{D_{i}^{t}(x^{t},y^{t})}\times \sqrt{\frac{D_{i}^{t}(x^{t+1},y^{t+1})}{D_{i}^{t+1}(x^{t+1},y^{t+1})}\times \frac{D_{i}^{t}(x^{t},y^{t})}{D_{i}^{t+1}(x^{t},y^{t})}}.$$

Some scholars further decomposed Effch into Pech and Sech combined with VRS, expressed as in Equation (4):

(4) $$\begin{array}{cc} \; & M_{i}\left(x^{t+1},y^{t+1};x^{t},y^{t}\right)\\ \; & \;=\;\frac{D_{i}^{t+1}\left(x^{t+1},y^{t+1}\left|VRS\right.\right)}{D_{i}^{t}\left(x^{t},y^{t}\left|VRS\right.\right)}\;\times \;\left[\frac{D_{i}^{t}\left(x^{t},y^{t}\left|VRS\right.\right)}{D_{i}^{t}\left(x^{t},y^{t}\left|CRS\right.\right)}/\frac{D_{i}^{t+1}\left(x^{t+1},y^{t+1}\left|VRS\right.\right)}{D_{i}^{t+1}\left(x^{t+1},y^{t+1}\left|CRS\right.\right)}\;\right]\;\times \;\left[\frac{D_{i}^{t}\left(x^{t},y^{t}\left|CRS\right.\right)}{D_{i}^{t+1}\left(x^{t},y^{t}\left|CRS\right.\right)}/\frac{D_{i}^{t}\left(x^{t+1},y^{t+1}\left|CRS\right.\right)}{D_{i}^{t+1}\left(x^{t+1},y^{t+1}\left|CRS\right.\right)}\;\right]\\ \; & \;=\;\mathrm{Techch}\;\times \;\mathrm{Sec}h\;\times \;\mathrm{Pech}\;=\;\mathrm{Tfpch} \end{array}$$

The subscript with VRS is a case of variable returns to scale, while the subscript with CRS is a case of constant returns to scale. The three items on the right side of Equation (4) are the technical efficiency change, scale efficiency change, and technical change under the condition of variable returns to scale [42]. Pech > 1 shows that the technical management level of SCC pilots has improved; if Sech > 1, the DMU is gradually getting closer to the optimal scale. Moreover, Tfpch > 1 shows an improvement in total efficiency; Tfpch = 1 indicates that the total efficiency is unchanged, while Tfpch < 1 means a reduction in total efficiency [41].

### 2.2. Index System for Efficiency Evaluation of SCC Pilots

SCC is a complex system project. In the selection of the indicators affecting SCC effectiveness, it is necessary to comprehensively consider the economic, social, and natural ecological factors that affect SCC. In terms of economic and social factors, the efficiency of SCC is closely related to its economy, infrastructure, and population. The construction of different sponge cities varies according to the economy, infrastructure, and population. National SCC pilots are affected by factors such as the economic level, population scale, and hydrological conditions of each city, resulting in different economic development levels, planning goals, and sponge projects among pilots. Therefore, it is unscientific to evaluate efficiency only by the scale of SCC; instead, it is necessary to make a horizontal comparison based on the efficiency of the pilots [44]. According to the influencing factors of SCC and the actual output of urban construction, the efficiency of SCC in several pilots is appropriately evaluated. At the level of natural ecological factors, the differences in the background conditions of the environment between pilots will lead to different efficiency levels of SCC. For example, the topography and terrain of the pilot play an important role in the storage and discharge of rainwater, the climate characteristics and geographical location affect the precipitation and vegetation characteristics of the pilot, and the performance of urban infiltration facilities affects the soil permeability and groundwater level [45,46,47,48]. Considering the above contents, the constructed efficiency evaluation indicators system of SCC is shown in Table 1.

It can be seen from Table 1 that the indicators reflecting the urban economic level, population scale, and urbanization degree are selected as input indicators from the statistical yearbook of each pilot. Macro indicators of SCC are selected as output indicators, including urban forest coverage, centralized sewage treatment rate, per capita green land area, and greening coverage rate of built-up areas. Among the input indicators, the urban employed people reflects the population scale level of the pilot, and the employed population indirectly reflects the economic level of the pilot, which suggests the urban labor force and vitality; the population urbanization rate is an intuitive embodiment of urban urbanization; the gross domestic product of the pilot directly reflects the overall economic level of the city, which has an important impact on the infrastructure construction of a sponge city; and the development and utilization rate of water resources reflects whether the urban water resources reserve can meet the demand [49,50]. In terms of output indicators, a higher urban greening level, i.e., urban parks and forests, can ensure the capacity of SCC to retain water and perform water purification, which leads to the greater effectiveness of water ecological constructions. The urban domestic sewage treatment rate can directly reflect the water purification capacity of SCC and can indicate the effectiveness of the water environment of SCC. Therefore, the urban greening level, ecological infrastructure construction status, and urban sewage treatment capacity are direct indicators reflecting the performance of SCC, which can be used as output contents of the DEA model to evaluate the efficiency of DMUs. The indicator data in Table 1 are collected from the official statistical yearbook and water resources bulletin of each pilot. In addition, indicators such as the total annual runoff control rate and the density of the urban drainage pipe network can better reflect the ability to withstand urban rain and floods, but it is difficult to obtain the indicator data. Therefore, this paper mainly considers the water environment dimension of SCC.

### 2.3. Selection of SCC Pilots

According to the representativeness of the pilots and the availability of panel data, 11 national SCC pilots in Chongqing, Wuhan, Chizhou, Shanghai, Shenzhen, Xining, Beijing, Xiamen, Jinan, Hebi, and Sanya were selected as samples for analysis. In the selection of the announced SCC pilots, the urban area, geographical features, economic development level, etc., were considered, which makes the case analysis comparable and valuable [51,52]. The list of specific pilots is shown in Table 2.

The selected SCC pilots, with different geographical locations and geographical characteristics, have comparative value. Before applying to build an SPC, it is necessary to conduct a self-examination of the economic level of the city, design and propose a reasonable construction model, and ensure the high efficiency of construction and operation throughout the life cycle. In addition, according to the aim of “adopting a replicable and scalable innovation model such as technology plus capital and overall packaging” in the “Sponge City Construction Pilot City Application Guide” [9,53,54], it can be explained that the economic level and policy support of the pilots are meant to maintain the efficiency of urban-related projects. On top of that, after the pilot cities were determined in 2015 and 2016, each began to implement SCC projects according to its urban economic level, ecological and natural conditions, and the application of low-impact development technology. The construction period of a pilot city is three years, and the pilots conducted performance evaluations in 2018 and 2019. Considering the lag of the implementation effect of construction projects, the four-year urban panel data from 2017 to 2020 were selected as the basis for the dynamic efficiency evaluation of the pilots, and the specific year data status is shown in Table A1 and Table 3. Therefore, the selection of these 11 pilots can reflect the relative efficiency level of SCC to a great extent.

## 3. Results and Analysis

### 3.1. Static Analysis

**Figure 2:**
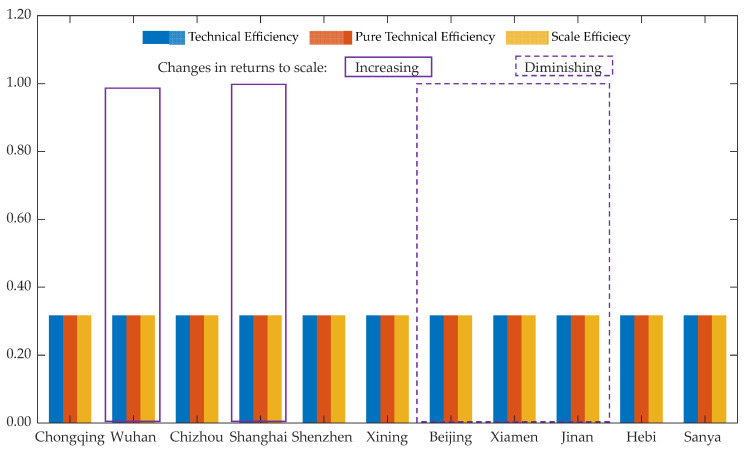
The efficiency of DMUs.

For the static efficiency evaluation of the SCC pilots, the urban panel data of each pilot in 2019 were selected. Because the construction period of the national SCC pilot is three years and all the pilots’ performances in 2019 were fully assessed [49,55], urban data from 2019 can reflect the cumulative long-term effect of urban water environment construction since the SCC pilot started running. In addition, it can eliminate the interference of the public health emergency (COVID-19) in 2020 on construction efficiency. Therefore, the results obtained by inputting the indicator data of each pilot city in 2019 into DEAP2.1 software (University of New England, Armidale, Australia) are shown in Figure 2.

According to Figure 2, the results of the comprehensive efficiency, pure technical efficiency, and scale efficiency of Chongqing, Chizhou, Xining, Hebi, and Sanya are all 1.000, indicating that DEA is effective in these pilots. The pure technical efficiency of Shenzhen, Beijing, Xiamen, and Jinan is equal to 1.000, which means these pilots need neither a corresponding input decrease nor an output increase, and the pure technical efficiency is effective. However, the scale of these pilots does not match the input and output, the scale efficiency is below 1.000, and the construction scale needs to be increased or reduced. Therefore, the overall comprehensive efficiency of those pilots is less than 1.000, and the overall DEA is invalid. The pure technical efficiency and scale efficiency of Wuhan and Shanghai are both less than 1.000, which are DEA-ineffective. It can be seen from Figure 1 that most cities have the problem of ineffective scale. Shenzhen, Beijing, Xiamen, and Jinan are effective in terms of pure technical efficiency, but the scale efficiency is invalid. Their input scales are redundant, resulting in low scale efficiency and decreasing returns to scale. From the perspective of fixing output, their input scale should be reduced. From a practical point of view, the reasons for the low scale efficiency of these cities should be explained from the perspective of output. A high level of input indicators, such as urban population quality and economic level, leads to a low level of output content, which results in the relative ineffectiveness of their scale efficiency.

According to the calculation rules of the DEA model and the calculation results of DEAP2.1 software, the improvement values of Wuhan and Shanghai in this model are compared based on the input indicators of Chizhou. When the scale efficiency factor is ignored and the technical efficiency of the decision unit is considered alone, it is necessary to compare the level of each indicator relative to the overall change. The calculation equation of the improvement rate is as follows. The results are shown in Table 4.

(5) $$\mathrm{Improvement}\;\mathrm{rate}=\frac{\mathrm{Radial}\;\mathrm{improvement}\;\mathrm{value}+\mathrm{Variable}\;\mathrm{improvement}\;\mathrm{value}}{\mathrm{Initial}\;\mathrm{value}}\times \;100\;\%,$$

where the radial improvement value refers to the value of the relaxation variable of the input index, that is the input redundancy value; the slack variable improvement value refers to the value of the slack variable of the output index, that is the value of insufficient output; and the target value is the value that achieves DEA effectiveness.

The lower pure technical efficiency of the two cities is caused by the lower output, and there are technical and policy problems in the planning and construction of the sponge city. Wuhan, in particular, has a pure technical efficiency of only 0.684, significantly lower than the other cities. Its relatively high urbanization rate input restrains the city’s SCC efficiency to a rather low level, thus making its pure technical efficiency lower. In addition, different from Shenzhen, Beijing, Xiamen, and Jinan with decreasing returns to scale, Wuhan and Shanghai are not fully effective in terms of scale efficiency, but the two cities have increasing returns to scale. This shows that if the overall scale of the two cities increases, the returns to scale will also increase, and the two cities still have great potential for the development of SCC. The accelerated urbanization of Wuhan is the main reason for the gradual loss of the sponge body. The reason why the efficiency of Wuhan and Shanghai is lower than that of Chizhou is that their input–output ratio is relatively poor. In order to achieve higher efficiency, the inputs need to be improved as shown in Table 4. The improvement rate is the increase or decrease rate from the initial value to the target value. The improvement rate of urban employed people and gross domestic product is large, while the improvement rate of population urbanization is small.

### 3.2. Dynamic Analysis

**Figure 3:**
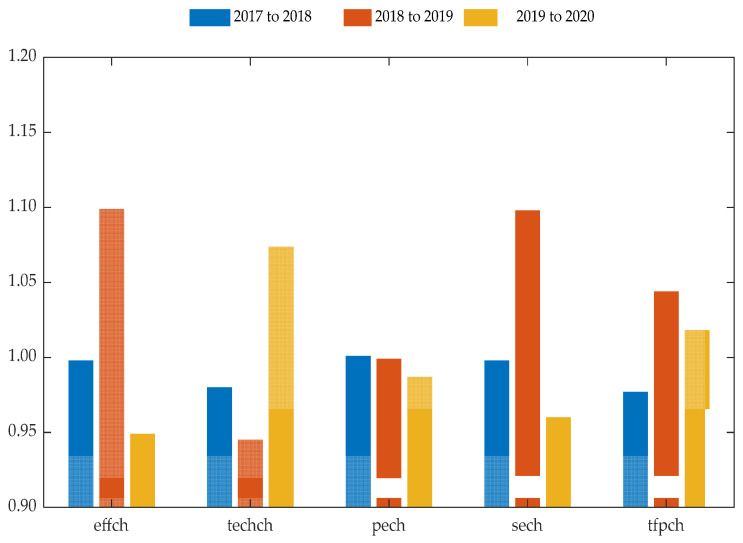
Change trend of average efficiency of the three-stage period.

In order to further analyze the urban construction efficiency of the pilots since the implementation of the national SCC pilot policy, the dynamic efficiency of the pilots was calculated via the Malmquist index model. The data from the 11 pilots from 2017 to 2020 were inputted into DEAP2.1 software, and the results are shown in Table 5 and Figure 3.

From a global perspective, according to the results of the Malmquist index model, the increase in total factor productivity of SCC in the 11 pilots mainly depends on the scale efficiency change index, which is limited by the technological progress index and the pure technical efficiency change index. From 2017 to 2018, only three of the selected pilots were in the growth state, with a total factor productivity index greater than 1 (accounting for only 27.27%); from 2018 to 2019, the proportion of the pilots in the growth state of the total factor productivity index was 45.46%; from 2019 to 2020, the pilots with a total factor productivity index in the growth state accounted for 54.55%; from 2017 to 2020, the average value of the total factor productivity index of all selected pilot cities was 1.013, which is greater than 1, so the overall efficiency of the SCC pilots was developing positively, which verifies that the overall SCC pilot policy in China exhibits environmentally friendly, positive, and sustainable development.

From a spatial perspective, in the two stages of 2017–2018 and 2018–2019, the total factor productivity index of Chongqing, Chizhou, Hebi, Xining, and the other pilots was in decline due to the influence of technological progress. In the initial stage of SCC, the urban economic level, population, and urbanization affect the scale of urban pilot projects’ construction, thus resulting in a relatively low efficiency of scale construction in some pilots with an inferior economic level and low population. The reason for that is the insufficient utilization of input factors in the pilots, which leads to resource redundancy due to the underutilization and transformation of some inputs. Therefore, the returns to scale cannot be formed—that is, the effect of the SCC is not optimal. From 2019 to 2020, the efficiency of the pilots changed significantly. Compared with the previous two stages, the total factor productivity index of Shanghai, Beijing, Jinan, and other cities decreased significantly, and the decline rate of some cities was higher than 0.30. There was a certain decline in scale and technical factors, while the decline rate of the technical progress index was not obvious. In the initial stage of the SCC pilot, the increase in total factor productivity in SCC depends on the change index of scale efficiency, which is affected by the level of urban economic development. From 2019 to 2020, the urban scale efficiency was in a state of decline, and the technological progress and pure technical efficiency had a relatively smooth change. The scale efficiency fluctuated greatly, and the scale efficiency was no longer dominated by the economic level. 

In terms of the time dimension, the mean value of the total factor productivity index of pilots in the three stages (2017–2018, 2018–2019, and 2019–2020) was 1.013; the mean value of the total factor productivity index of pilots in 2017–2018 was 0.977, while the mean value of the 2018–2019 and 2019–2020 phases was 1.044 and 1.018, respectively. From the perspective of the change in the three stages, the average value of the stage from 2017 to 2018 was less than 1. Due to the technological progress index level and pure technical efficiency of the pilots being relatively low, the average level of the total factor productivity efficiency was low. From 2018 to 2019, the scale index and the total factor productivity index were increasing, while the technological progress index remained at a standstill, but the pure technological change index was in an increasing state. From 2019 to 2020, due to the expiration of the pilot construction period and the COVID-19 pandemic, the change index of scale efficiency decreased, but the total factor growth index was still greater than 1, remaining in a state of growth. From 2017 to 2020, the overall total factor productivity efficiency gradually and steadily increased. The inputs of urban economy, population, and other factors were fully transformed, and the technological level of the low-impact development of sponge cities gradually increased, making the scale of water environment construction more effective. Under the same scale conditions, the returns to scale increased. In the initial stage of SCC, the efficient construction of an urban construction scale leads to positive development in SCC. During the construction process, technological progress leads to a steady growth in total factor productivity; even if the scale efficiency is affected by emergencies, the total factor productivity is still in a growing state.

## 4. Conclusions

This paper selects the macro indicators that reflect the outputs of SCC as the outputs of efficiency evaluation, which can comprehensively reflect the efficiency of SCC pilots. The efficiency evaluation model of SCC is established by using the DEA method, which not only reflects the relative efficiency of the input and output of SCC via the relative efficiency value of DMUs, but also analyzes the direction and scale of urban construction according to the pure technical efficiency and the scale efficiency of DMUs. The DEA model for efficiency evaluation of SCC could be used to evaluate the efficiency of pilots and obtained some valuable results.

From a static perspective, most SCC pilots are fully effective, while a few pilots are ineffective. Among them, the pure technical efficiency of Wuhan is the lowest (0.684); the scale efficiency of Xiamen is the lowest (0.626), resulting in the lowest comprehensive efficiency of 0.626. The pure technical efficiency of Wuhan and Shanghai is low, which leads to a technical efficiency less than 1; Shenzhen and Xiamen have low scale efficiency, which leads to a technical efficiency less than 1. The SCC efficiency of Chizhou, Chongqing, and other pilots is fully effective—that is, under the assumption of a variable scale, the pure technical efficiency and scale efficiency are both effective.

From a dynamic perspective, from 2017 to 2020, the average total factor productivity of all pilot cities was 1.013. This shows that the overall efficiency in the selected 11 SCC pilot cities was good. From 2017 to 2018, the average total factor productivity of the pilot cities was 0.977, mainly because the comprehensive technical efficiency change index was less than 1; from 2018 to 2019, the comprehensive technical efficiency change index was 1.099, and the average total factor productivity of the pilot cities increased to 1.044; from 2019 to 2020, the average total factor productivity of the pilot cities was 1.018, which was lower than the previous period but still greater than 1, mainly because the technological progress index changed to 1.074, which improved the total factor productivity.

## 5. Recommendations

According to the results of the model analysis and the current situation of SCC pilots in China, countermeasures and suggestions are put forward as follows.

1. SCC pilots should pay attention to the efficiency evaluation and speed up the establishment of the SCC efficiency evaluation system. The water environment problem is complex. In order to improve the efficiency of SCC, it is necessary to take into account the technicality of construction and the effectiveness of scale. The reason for the ineffectiveness of DEA in some pilots is due to the low pure technical efficiency and a certain input-to-output ratio problem. The efficiency evaluation of SCC is a dissection of the problems existing in terms of the technology and scale of the existing sponge city projects, and also a guide for future sponge city planning and construction. With the continuous promotion of the SCC pilots and the popularization of the concept of global SCC, sponge cities will be a key direction of urban ecological planning and construction in the future. At this stage, a relatively complete SCC efficiency evaluation system has not yet been constructed. Therefore, at this stage, accelerating the establishment of an SCC efficiency evaluation system is a key way to evaluate the effectiveness of SCC and to determine the difficulties of SCC, which is also an important part of the planning, construction, and future governance of sponge cities. We have mainly considered the efficiency of SCC from the perspective of the water environment. In later stages, rainwater and flood management dimension indicators, such as the length of drainage pipelines and the density of the drainage pipe network, should be added to make the efficiency evaluation system more scientific, reasonable, and comprehensive.
2. It is necessary to rationalize the investment structure and improve the scale efficiency of SCC. In the static analysis of 11 SCC pilots, only four were effective in terms of scale efficiency, and seven were ineffective, especially Shenzhen and Xiamen. Most SCC pilots with a low scale efficiency are characterized by decreasing returns to scale. Although the overall scale of the pilot is large and the urbanization rate is high, the green infrastructure investment, urban greening level, effective use of water resources, and other explicit sponge city indicators are difficult to match. Rapid urbanization conflicts with the high demands for urban infrastructure construction. Therefore, in the process of urban development, the government should pay attention to the construction of green infrastructure with the sponge city concept as the main body, to improve the water environment of the city. Ultimately, the government should build a sustainable development model that matches the scale of the city, the urban ecological environment, and the number of sponge bodies.
3. It is suggested that SCC pilots should improve technical efficiency and enhance the efficiency of all construction factors. According to the dynamic analysis of the Malmquist index, in the whole period from 2017 to 2020, the total factor productivity index was limited by the technological progress index. The results reflect a marginal decrease in the output of the productivity efficiency of input factors under the optimal input scale. Especially in the initial stage of SCC, the technological progress index and pure technical efficiency were limited by the growth of the total factor productivity index, which is the main factor of construction efficiency in the initial stage of sponge city construction and development. When cities set out to launch large-scale SCC pilot projects, the construction concept, which is a balance between technology and scale development, should be applied. In addition, when the scale efficiency of a city is impacted under special circumstances, the total factor productivity will also be greatly affected; therefore, the input structure should be improved according to the scale factors.

## Figures and Tables

**Table 1:** Efficiency evaluation indicators system of SCC.

Indicator Type	Indicator Name (Unit)	Indicator Calculation Formula
Input indicators (Urban population, economy, urbanization, water resources, and other macro indicators)	Urban employed people (10,000 persons)	—
	Gross domestic product (100 million Yuan)	—
	Development and utilization rate of water resources (%)	Total water consumption/Total water resources × 100%
	Population urbanization rate (%)	Urban resident population/Total resident population × 100%
Output indicators (Urban water ecological environment indicators)	Urban forest coverage (%)	Urban forest coverage/Total urban area × 100%
	Centralized sewage treatment rate (%)	Urban domestic sewage treatment capacity/Total urban domestic sewage discharge × 100%
	Per capita green land area (square meter)	Urban public park green space area/Urban population
	Greening coverage rate of built-up areas (%)	The vertical projected area of vegetation/Total area of urban land × 100%

**Table 2:** Geographic location and characteristics of the selected pilots.

Sponge City Pilot	Geographical Position	Urban Comprehensive Grade	Geographical Features
Chongqing	Southwest China	New first-tier city	The city has a mild climate, many low mountains and shallow hills, great potential for hydropower development, and uneven distribution of groundwater sources.
Wuhan	Central China	New first-tier city	The city is mainly flat; there are many lakes and ponds, with rich water resources.
Chizhou	Eastern China	Fourth-tier city	The city is high in the southeast and low in the northwest, with a ladder distribution from south to north, and is rich in water resources.
Shanghai	Eastern China	First-tier city	The city has a flat terrain, facing the sea in the east, abundant rainfall, and abundant water resources.
Shenzhen	Southern China	First-tier city	The city has many low hills and the central south coast, with abundant rainfall, but the rainwater collection area and flow are small, and the freshwater resources are relatively scarce.
Xining	Northwest China	Fourth-tier city	The city is rich in surface water and groundwater resources.
Beijing	Northern China	Fourth-tier city	The city is mainly plain, and freshwater resources are relatively scarce. Fresh water comes from outside the city.
Xiamen	Southeast China	Second-tier city	The southeast coast of the city is mild and rainy, and freshwater resources are relatively scarce.
Jinan	Eastern China	Second-tier city	The city has less rain, an obvious monsoon climate, and sufficient groundwater resources.
Hebi	Central China	Fifth-tier city	The city is located inland, with relatively scarce water resources and large water demands.
Sanya	Southern China	Third-tier city	The city is surrounded by the sea, and there is a lack of freshwater resources.

**Table 3:** Descriptive statistics of the SCC pilots’ main variables from 2017 to 2020.

Year	Variable	Mean	Max	Min	Std. Dev
2020	Urban forest coverage	42.394	69.000	18.500	206.716
	Centralized sewage treatment rate	97.114	100.400	93.000	4.684
	Per capita green land area	14.469	18.000	9.050	6.130
	Greening coverage rate of built-up areas	42.802	48.960	37.320	8.744
	Urban employed people	647.199	1676.010	65.900	379,294.137
	Gross domestic product	14,848.682	38,700.580	495.410	215,790,158.012
	Development and utilization rate of water resources	96.865	210.096	8.118	4367.614
	Population urbanization rate	75.183	99.540	59.680	165.158
2019	Urban forest coverage	40.326	69.000	17.560	180.558
	Centralized sewage treatment rate	95.358	97.730	91.410	4.378
	Per capita green land area	14.577	20.300	8.400	11.876
	Greening coverage rate of built-up areas	42.582	48.400	39.700	6.782
	Urban employed people	691.944	1704.540	54.900	391,840.863
	Gross domestic product	14,491.835	38,155.320	677.900	205,098,062.499
	Development and utilization rate of water resources	88.971	258.227	10.198	6283.075
	Population urbanization rate	70.592	99.520	54.900	195.817
2018	Urban forest coverage	39.540	68.500	16.900	242.209
	Centralized sewage treatment rate	93.655	97.600	77.350	32.074
	Per capita green land area	13.670	16.550	8.200	7.132
	Greening coverage rate of built-up areas	42.214	48.400	39.400	7.708
	Urban employed people	694.846	1909.510	54.900	419,908.134
	Gross domestic product	12,597.231	32,679.870	622.300	155,146,748.396
	Development and utilization rate of water resources	75.995	267.183	7.583	6693.965
	Population urbanization rate	73.385	99.750	54.100	233.461
2017	Urban forest coverage	37.742	69.000	15.020	277.837
	Centralized sewage treatment rate	93.155	96.810	80.720	26.112
	Per capita green land area	13.855	17.450	8.190	9.182
	Greening coverage rate of built-up areas	42.130	48.420	39.100	8.197
	Urban employed people	638.993	1659.330	52.750	381,417.451
	Gross domestic product	10,633.847	30,632.990	546.010	137,497,937.025
	Development and utilization rate of water resources	90.123	223.618	9.775	5412.078
	Population urbanization rate	75.355	99.000	53.670	192.632

**Table 4:** Input redundancy and target value of invalid DEA units.

Input Indicators	DMU	Initial Value	Radial Improvement Value	Slack Variable Improvement Value	Target Value	Improvement Rate (%)
Urban employed people (10,000 persons)	Wuhan	623.13	−197.05	−311.68	114.40	−81.64
	Shanghai	1376.20	−129.45	−1132.36	114.40	−91.69
Gross domestic product (100 million Yuan)	Wuhan	16,223.21	−5130.24	−10,261.27	831.70	−94.87
	Shanghai	38,155.32	−3588.87	−33,734.75	831.70	−97.82
Development and utilization rate of water resources (%)	Wuhan	49.39	−15.62	−14.67	19.10	−61.33
	Shanghai	157.19	−14.79	−123.30	19.10	−87.85
Population urbanization rate (%)	Wuhan	80.29	−25.39	0.00	54.90	−31.62
	Shanghai	60.60	−5.70	0.00	54.90	−9.41

**Table 5:** Malmquist index of pilots from 2017 to 2020.

Year	DMU	Effch	Techch	Pech	Sech	Tfpch
2017–2018	Chongqing	1.033	0.845	1.000	1.033	0.873
	Wuhan	1.048	0.975	1.000	1.048	1.022
	Chizhou	1.000	0.834	1.000	1.000	0.834
	Shanghai	1.008	1.000	1.006	1.003	1.008
	Shenzhen	0.996	0.994	1.000	0.996	0.990
	Xining	1.000	1.217	1.000	1.000	1.217
	Beijing	1.005	0.994	1.000	1.005	1.000
	Xiamen	0.903	0.999	1.000	0.903	0.903
	Jinan	0.985	1.000	1.000	0.985	0.984
	Hebi	1.000	0.949	1.000	1.000	0.949
	Sanya	1.000	0.968	1.000	1.000	0.968
2018–2019	Chongqing	1.020	0.924	1.000	1.020	0.943
	Wuhan	0.855	0.930	0.980	0.873	0.795
	Chizhou	1.000	0.775	1.000	1.000	0.775
	Shanghai	1.471	1.000	1.013	1.451	1.471
	Shenzhen	0.995	1.003	1.000	0.995	0.997
	Xining	1.000	0.832	1.000	1.000	0.832
	Beijing	1.420	1.005	1.000	1.420	1.427
	Xiamen	1.017	1.005	1.000	1.017	1.022
	Jinan	1.307	1.002	1.000	1.307	1.311
	Hebi	1.000	1.078	1.000	1.000	1.078
	Sanya	1.000	0.837	1.000	1.000	0.837
2019–2020	Chongqing	0.908	1.444	1.000	0.908	1.311
	Wuhan	1.089	0.939	0.991	1.099	1.023
	Chizhou	1.000	1.555	1.000	1.000	1.555
	Shanghai	0.892	0.947	0.965	0.924	0.844
	Shenzhen	1.083	0.927	0.987	1.097	1.004
	Xining	0.776	1.106	0.939	0.827	0.859
	Beijing	0.771	0.908	1.000	0.771	0.701
	Xiamen	1.111	0.946	1.000	1.111	1.051
	Jinan	0.806	0.969	0.978	0.824	0.781
	Hebi	1.000	0.993	1.000	1.000	0.993
	Sanya	1.000	1.081	1.000	1.000	1.081
Mean value		1.015	0.999	0.996	1.019	1.013

## Data Availability

Not applicable.

## Appendix A

**Table A1:** Original values of main variables in 11 pilots from 2017 to 2020.

**Variable/Chongqing**	**2017**	**2018**	**2019**	**2020**
Urban forest coverage (%)	46.50	48.30	50.10	52.50
Centralized sewage treatment rate (%)	94.70	94.91	94.91	97.95
Per capita green land area (square meter)	17.05	16.55	16.16	16.16
Greening coverage rate of built-up areas (%)	40.32	40.42	41.74	43.05
Urban employed people (10,000 persons)	1659.33	1909.51	1704.54	1676.01
Gross domestic product (100 million Yuan)	6039.4	20,363.19	23,065.77	25,002.79
Development and utilization rate of water resources (%)	11.80	14.72	15.35	9.14
Population urbanization rate (%)	65.00	65.50	66.80	69.46
**Variable/Wuhan**	**2017**	**2018**	**2019**	**2020**
Urban forest coverage (%)	15.02	22.88	42.07	42.07
Centralized sewage treatment rate (%)	96.00	96.47	95.06	97.00
Per capita green land area (square meter)	9.62	9.65	10.19	14.04
Greening coverage rate of built-up areas (%)	39.55	39.46	40.02	42.07
Urban employed people (10,000 persons)	564.08	610.72	623.13	603.79
Gross domestic product (100 million Yuan)	13,090.81	14,847.29	16,223.21	15,616.1
Development and utilization rate of water resources (%)	88.49	16.13	49.40	108.98
Population urbanization rate (%)	72.60	73.20	80.29	80.49
**Variable/Chizhou**	**2017**	**2018**	**2019**	**2020**
Urban forest coverage (%)	59.75	59.90	44.90	60.40
Centralized sewage treatment rate (%)	94.46	95.20	96.60	95.80
Per capita green land area (square meter)	17.45	15.42	15.50	16.70
Greening coverage rate of built-up areas (%)	43.40	43.50	44.37	43.80
Urban employed people (10,000 persons)	114.47	114.57	114.40	65.90
Gross domestic product (100 million Yuan)	624.35	684.90	831.70	868.90
Development and utilization rate of water resources (%)	11.23	14.98	19.10	8.12
Population urbanization rate (%)	53.67	54.10	54.90	59.68
**Variable/Shanghai**	**2017**	**2018**	**2019**	**2020**
Urban forest coverage (%)	16.20	16.90	17.56	18.50
Centralized sewage treatment rate (%)	93.99	95.20	96.30	96.17
Per capita green land area (square meter)	8.19	8.20	8.40	9.05
Greening coverage rate of built-up areas (%)	39.10	39.40	39.70	37.32
Urban employed people (10,000 persons)	1372.65	1375.66	1376.2	1374
Gross domestic product (100 million Yuan)	30,632.99	32,679.87	38,155.32	38,700.58
Development and utilization rate of water resources (%)	223.62	267.18	157.19	166.38
Population urbanization rate (%)	87.70	88.10	60.60	71.69
**Variable/Shenzhen**	**2017**	**2018**	**2019**	**2020**
Urban forest coverage (%)	40.00	39.80	39.80	39.39
Centralized sewage treatment rate (%)	96.81	97.60	97.72	98.00
Per capita green land area (square meter)	15.95	15.40	14.90	15.00
Greening coverage rate of built-up areas (%)	45.10	45.00	43.00	43.40
Urban employed people (10,000 persons)	1252.83	1291.31	1283.37	1292.29
Gross domestic product (100 million Yuan)	22,490.06	24,221.98	26,927.09	27,670.24
Development and utilization rate of water resources (%)	102.96	89.80	77.37	103.38
Population urbanization rate (%)	99.00	99.75	99.52	99.54
**Variable/Xining**	**2017**	**2018**	**2019**	**2020**
Urban forest coverage (%)	33.00	34.00	35.10	36.00
Centralized sewage treatment rate (%)	85.67	91.41	91.41	93.00
Per capita green land area (square meter)	12.47	12.30	12.50	12.82
Greening coverage rate of built-up areas (%)	40.50	40.50	40.58	40.50
Urban employed people (10,000 persons)	131.93	132.84	133.21	134.50
Gross domestic product (100 million Yuan)	1284.91	1260.86	1363.59	1372.98
Development and utilization rate of water resources (%)	9.78	7.58	10.20	49.23
Population urbanization rate (%)	71.14	59.58	72.85	73.00
**Variable/Beijing**	**2017**	**2018**	**2019**	**2020**
Urban forest coverage (%)	35.84	43.50	44.00	43.77
Centralized sewage treatment rate (%)	94.98	93.40	95.00	94.76
Per capita green land area (square meter)	16.20	16.30	16.40	16.59
Greening coverage rate of built-up areas (%)	48.42	48.40	48.40	48.96
Urban employed people (10,000 persons)	1246.8	1237.8	1397.4	1164
Gross domestic product (100 million Yuan)	29,883	30,320	35,371.3	36,102.6
Development and utilization rate of water resources (%)	132.55	110.70	169.79	157.36
Population urbanization rate (%)	86.50	86.50	60.60	87.50
**Variable/Xiamen**	**2017**	**2018**	**2019**	**2020**
Urban forest coverage (%)	42.80	43.00	43.80	44.00
Centralized sewage treatment rate (%)	95.77	96.02	96.36	100.00
Per capita green land area (square meter)	14.09	14.85	15.60	14.60
Greening coverage rate of built-up areas (%)	43.59	44.10	45.13	45.52
Urban employed people (10,000 persons)	146.00	308.30	334.48	126.70
Gross domestic product (100 million Yuan)	4351.18	4791.41	6384	6384.02
Development and utilization rate of water resources (%)	76.15	44.79	62.26	127.82
Population urbanization rate (%)	89.10	89.10	89.20	89.41
**Variable/Jinan**	**2017**	**2018**	**2019**	**2020**
Urban forest coverage (%)	24.45	25.56	25.56	27.40
Centralized sewage treatment rate (%)	95.98	96.59	97.73	98.17
Per capita green land area (square meter)	11.79	13.30	20.30	12.80
Greening coverage rate of built-up areas (%)	40.57	40.52	41.18	40.70
Urban employed people (10,000 persons)	405.38	510.30	492.36	510.30
Gross domestic product (100 million Yuan)	7201.96	7856.56	9443.4	10,140.91
Development and utilization rate of water resources (%)	155.70	79.85	124.13	85.00
Population urbanization rate (%)	70.53	72.10	71.21	73.46
**Variable/Hebi**	**2017**	**2018**	**2019**	**2020**
Urban forest coverage (%)	32.60	32.60	31.70	33.30
Centralized sewage treatment rate (%)	95.62	96.05	96.05	97.00
Per capita green land area (square meter)	14.19	14.20	18.00	18.00
Greening coverage rate of built-up areas (%)	39.91	41.95	43.19	43.20
Urban employed people (10,000 persons)	82.70	97.40	97.40	101.42
Gross domestic product (100 million Yuan)	827.65	921.18	966.90	980.97
Development and utilization rate of water resources (%)	163.77	171.04	258.23	210.10
Population urbanization rate (%)	58.76	60.07	61.31	62.51
**Variable/Sanya**	**2017**	**2018**	**2019**	**2020**
Urban forest coverage (%)	69.00	68.50	69.00	69.00
Centralized sewage treatment rate (%)	80.72	77.35	91.80	100.40
Per capita green land area (square meter)	15.41	14.20	12.40	13.40
Greening coverage rate of built-up areas (%)	42.97	41.10	41.10	42.30
Urban employed people (10,000 persons)	52.75	54.90	54.90	70.28
Gross domestic product (100 million Yuan)	546.01	622.30	677.90	695.41
Development and utilization rate of water resources (%)	15.29	19.17	35.66	40.00
Population urbanization rate (%)	74.91	59.23	59.23	60.27
